# Motor cortex electrical stimulation augments sprouting of the corticospinal tract and promotes recovery of motor function

**DOI:** 10.3389/fnint.2014.00051

**Published:** 2014-06-18

**Authors:** Jason B. Carmel, John H. Martin

**Affiliations:** ^1^Department of Neurology, Weill Cornell Medical CollegeNew York, NY, USA; ^2^Department of Pediatrics, Weill Cornell Medical CollegeNew York, NY, USA; ^3^Brain and Mind Research Institute, Weill Cornell Medical CollegeNew York, NY, USA; ^4^Burke Medical Research InstituteWhite Plains, NY, USA; ^5^Department of Physiology, Pharmacology and Neuroscience, City College of the City University of New YorkNew York, NY, USA

**Keywords:** motor cortex, corticospinal tract, activity-dependent sprouting, locomotion, rehabilitation

## Abstract

The corticospinal system—with its direct spinal pathway, the corticospinal tract (CST) – is the primary system for controlling voluntary movement. Our approach to CST repair after injury in mature animals was informed by our finding that activity drives establishment of connections with spinal cord circuits during postnatal development. After incomplete injury in maturity, spared CST circuits sprout, and partially restore lost function. Our approach harnesses activity to augment this injury-dependent CST sprouting and to promote function. Lesion of the medullary pyramid unilaterally eliminates all CST axons from one hemisphere and allows examination of CST sprouting from the unaffected hemisphere. We discovered that 10 days of electrical stimulation of either the spared CST or motor cortex induces CST axon sprouting that partially reconstructs the lost CST. Stimulation also leads to sprouting of the cortical projection to the magnocellular red nucleus, where the rubrospinal tract originates. Coordinated outgrowth of the CST and cortical projections to the red nucleus could support partial re-establishment of motor systems connections to the denervated spinal motor circuits. Stimulation restores skilled motor function in our animal model. Lesioned animals have a persistent forelimb deficit contralateral to pyramidotomy in the horizontal ladder task. Rats that received motor cortex stimulation either after acute or chronic injury showed a significant functional improvement that brought error rate to pre-lesion control levels. Reversible inactivation of the stimulated motor cortex reinstated the impairment demonstrating the importance of the stimulated system to recovery. Motor cortex electrical stimulation is an effective approach to promote spouting of spared CST axons. By optimizing activity-dependent sprouting in animals, we could have an approach that can be translated to the human for evaluation with minimal delay.

## INTRODUCTION

Paralysis can be viewed as a disconnection between the brain circuits that initiate movement and the spinal cord centers that execute movement. In humans, the primary system controlling voluntary movement, the corticospinal system, is also the system most responsible for loss of function when it is injured. Given its importance in health and disease, the corticospinal tract (CST), which directly connects motor cortex to the spinal cord, has been a prime target for injury and repair studies.

Two paradigms have informed our approach to CST repair: the development of CST spinal connections and CST plasticity induced by partial injury. During development, activity is a critical determinant of CST connectivity, particularly at the level of synapses onto spinal cord targets (Martin et al., 2009; Friel et al., 2013). After partial injury in maturity, spared CST circuits sprout and restore lost function. Each of these processes is likely based on a similar competition for local trophic factors (Singh and Miller, 2005; Ueno et al., 2012). In the case of development, active CST connections to the spinal cord outcompete the less active ones. In the case of injury, axons sprout because a major source of competition has been lost due to the injury.

In this review, we describe the basic organization of the corticospinal system and its development. We summarize our discoveries about the role of activity in development, and how they inform our approach to repair of the injured system in mature animals using electrical stimulation. We demonstrate how we use electrical stimulation of spared CST connections after injury to augment the endogenous response to injury by increasing sprouting of spared CST connections. After summarizing our understanding of the effects of electrical stimulation on CST circuits and behavioral recovery, we end by discussing the implications of our studies for people living with paralysis.

## CORTICOSPINAL MOTOR CONTROL

The corticospinal system is the principal pathway by which we turn thoughts into actions. As the only cortically based motor path, the CST is highly adapted for limb control; hand and forelimb control in particular. The size of the CST and its functional importance correlate highly with forelimb or hand dexterity (Iwaniuk et al., 1999). As a descending motor system originating in cortex, the CST has access to sensory input and the internal framework from which a motor plan is derived. Control signals are then sent to execute that plan. The large loss of function that accompanies CST injury underscores its importance. In stroke (Stinear et al., 2007; Lindenberg et al., 2012) and spinal cord injury (Raineteau and Schwab, 2001), the degree of CST injury strongly predicts the resulting motor impairment.

**FIGURE 1:**
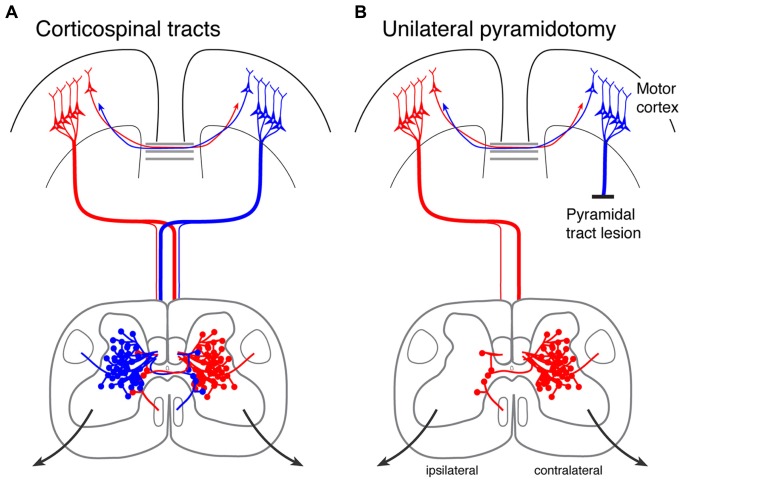
**Corticospinal tracts in the intact rodent and after unilateral pyramidotomy. (A)** The corticospinal tracts originating in each hemisphere are shown, together with their callosal interconnections. The corticospinal tract axons descend in the dorsal, lateral, and ventral columns; most descend in the dorsal columns. Note that each hemisphere gives rise to dense contralateral and sparse ipsilateral spinal terminations. **(B)** Unilateral pyramidal tract lesion (pyramidotomy) eliminates all corticospinal tract axons from one hemisphere. What remains on the affected side are sparse terminations from the ipsilateral motor cortex.

The CST is a primarily crossed pathway, with 80–95% of the axons terminating on the contralateral side (**Figure 1**). The sparse ipsilateral termination largely reflects “double crossing” of CST axons, first in the pyramid and then in the segmental spinal cord, as well as some axons that descend ipsilaterally in the white matter (Rosenzweig et al., 2009). While sparse in the rodent (Brosamle and Schwab, 2000), the ipsilateral CST is more robust in primates (Rosenzweig et al., 2009), especially to the cervical spinal cord, suggesting that the ipsilateral CST is a good target for promoting its connections. The CST is the direct spinal path from motor cortex; there are also indirect paths that relay in brain stem motor centers, especially the red nucleus and the reticular formation (Jankowska and Edgley, 2006).

Given that each motor cortex sends projections to both halves of the spinal cord, we studied how each forelimb is represented in the motor cortex of one hemisphere. Understanding this organization is important for reestablishing motor control after injury to one hemisphere: one could try to strengthen preserved connections from the injured hemisphere or create control of both sides of the body from the uninjured hemisphere. Using microstimulation mapping, we found that the representation of the forelimb ipsilateral to motor cortex stimulation is remarkably robust in the intact rat (Brus-Ramer et al., 2009). Less than 5% of CST axons projecting from motor cortex terminate in the ipsilateral half of the spinal cord in the rat (Brosamle and Schwab, 1997). Yet microstimulation provoked movement in the ipsilateral forelimb in about half of the sites in which contralateral responses were found. The types of movements, whether proximal or distal, were similar for the ipsilateral and contralateral forelimbs. However, we found that the microstimulation currents needed to evoke an ipsilateral movement are 2.4 times greater than those required to produce a contralateral movement (Brus-Ramer et al., 2009). These findings suggest that the ipsilateral CST, though sparse, has properties that could help to restore function after significant injury to the CST from the other hemisphere.

Our findings fit with substantial evidence for an adaptive role of the ipsilateral CST in motor control. In humans, the ipsilateral hemisphere is recruited during difficult tasks in health (van den Berg et al., 2011) and after stroke to the other hemisphere (Grefkes and Ward, 2014). In addition, stroke of one hemisphere causes impairment of the ipsilateral hand (Noskin et al., 2008), although it is less severe than the contralateral impairment. Tadashi Isa and colleagues, using a primate model of unilateral CST injury, have demonstrated that the ipsilateral activation is also necessary for the acute phase of recovery. Blocking motor cortex activity on the side without CST injury using the GABA agonist muscimol caused the initial motor recovery to be lost (Nishimura et al., 2007). By contrast, injury of the CST in rats does not cause a measurable decline in ipsilateral motor performance (Whishaw and Metz, 2002), indicating a diminished role of the ipsilateral CST in motor function in rodents. Our approach, as discussed below, is to harness activity-dependent processes to augment the density and strength of ipsilateral CST projections. The goal of this intervention is to promote the representation of the ipsilateral forelimb in the spared motor cortex, and by doing so, it could take on a significant role in ipsilateral limb control after injury to the other hemisphere.

## ROLE OF ACTIVITY IN CST DEVELOPMENT

Establishment of CST connections with spinal targets depends on an interplay between axon guidance molecules, CST neuron activity, and limb use. A key insight from our developmental studies is that competition between more and less active systems is important (Martin et al., 2009; Friel et al., 2013). Augmenting and blocking CST activity during development have opposite effects on establishment of spinal connections. Unilateral electrical stimulation of the CST during early postnatal development leads to a remarkable expansion of the contralateral and ipsilateral terminal fields (Salimi and Martin, 2004; Salimi et al., 2008). This expansion is partly due to maintaining transient terminations, since aberrant regions innervated by the CST early in development continue to be innervated after stimulation, as well as CST axonal outgrowth into novel territories. Blockade of CST activity, by muscimol infusion directly into motor cortex, leads to a robust reduction in the termination space of the inactivated CST (Friel and Martin, 2007). Moreover, there is a motor impairment contralateral to the inactivated motor cortex. Restricting limb use during early postnatal life also has a particularly robust effect on CST axon terminal morphology and function; effects that are similar to those produced by motor cortex inactivation (Martin et al., 2007). The changes after inactivation, as well as limb disuse, are permanent without further intervention.

We also discovered that, whereas absolute activity levels may be important during development, the relative level of activity between the CS systems from each hemisphere is key (Martin and Lee, 1999; Friel and Martin, 2007) Bilateral motor cortex inactivation has a muted effect on the pattern of CST development compared with unilateral inactivation. Further, unilateral activation of the CST results in a significant reduction in the CST projection from the non-activated side (Friel and Martin, 2007). Together these findings point to the importance of activity-dependent competition in shaping the establishment of CST connections in the spinal cord. To summarize, by limiting activity CST axons fail to grow to the usual targets and fail to develop normal branching and presynaptic sites. By augmenting activity, there is robust spinal axonal outgrowth. Importantly, activity-based interventions can be used in maturity in the cat to augment CST outgrowth, suggesting that CST neurons do not have an early developmental critical period for axonal remodeling – for both elimination and new local branch formation (Friel et al., 2012).

## MOST INJURIES SPARE CST AXONS, WHICH SPROUT AND RESTORE FUNCTION

The biological process that most strongly correlates with endogenous functional recovery is axon sprouting. Sprouting is defined as outgrowth from uninjured axons, either from uninjured pathways or proximally from axotomized neurons (Dietz and Fouad, 2014). This is in contrast to axon regeneration, which is outgrowth at the site of axotomy. Sprouting has been documented at the penumbra of cortical strokes and at subcortical targets of the CST. The Tuszynski group has documented CST sprouting after spinal cord injury in the rat and the monkey. After hemisection of the spinal cord in macaque, the CST from the uninjured half of the spinal cord sprouts profusely onto the denervated half of the cord. This sprouting is estimated to reconstitute 60% of the innervation of the intact spinal cord, and the robust sprouting correlates strongly with functional recovery (Rosenzweig et al., 2010). In the rat, after partial spinal cord injury that interrupts over 90% of CST axons, the spared CST fibers also sprout profusely. This sprouting also correlates with functional recovery. Injury of some remaining CST axons (as well as other motor fibers) abrogates the endogenous recovery after injury. There is tremendous capacity for spontaneous recovery after brain and spinal cord injury in people, as well, and this recovery may also be due to sprouting (Ruber et al., 2012).

Activity-based neurorehabilitation may target sprouting of spared connections (Tuszynski and Steward, 2012). Most brain and spinal cord injuries spare motor and sensory circuits that can be used to restore function in paralyzed limbs (Hagg, 2006). In the case of focal brain injuries, including stroke, the spared regions include perilesional cortex on the injured side (Jankowska and Edgley, 2006) and connections from the uninjured hemisphere that can control the paretic side of the body (Overman and Carmichael, 2014). In the case of spinal cord injury, most people have injuries that spare some movement or sensation below the injury site (Devivo, 2012). Even people without movement or sensation below the lesion site, there are usually anatomical connections bridging the injury site that can become functional (Bunge et al., 1997).

Whereas spontaneous motor recovery after injury may be partly mediated by plasticity in spared systems – for example, by injury-dependent CST sprouting – this plasticity alone is insufficient to return significant motor function after a serious injury. It is not clear why the capacity for functional recovery is limited. One explanation is that there are too few spared axons/connections. This receives support from neuropathological studies, as well as functional studies in humans showing elevated transcranial magnetic stimulation (TMS) thresholds to produce motor responses or even no evoked responses (Reis et al., 2008). Also, many movement parameters are coded at the population level in motor cortex. If this involves CST neurons, then whittling down the population by injury should lead, at some critical threshold, to impairment. Another potential reason for limited recovery is the loss of excitatory drive to spinal motor circuits that would accompany profound loss of descending excitatory inputs.

To promote motor function after SCI will thus require a larger substrate of descending connections to drive diverse functions of the largely denervated spinal motor circuits. Promoting sprouting of spared motor pathway axons is an important and effective strategy. This is because spared axons are present at segmental levels after injury. Spared axons need only sprout a short distance to contact their motor circuit targets, segmental interneurons, propriospinal/intersegmental interneurons, and motoneurons. CST sprouts can grow extensively within both the immature and the mature spinal gray matter, and even across the midline (Brus-Ramer et al., 2007; Rosenzweig et al., 2010). Thus, there is a largely untapped opportunity to promote recovery by promoting CST sprouting (Hagg, 2006).

## A METHODOLOGY TO ACTIVATE THE CORTICOSPINAL MOTOR SYSTEM IN MATURE ANIMALS

Our developmental findings suggested to us that manipulating activity of the corticospinal system could promote formation of connections after injury in mature animals. We developed a rat model to test the use of activity to promote sprouting of spared CST axons after injury (Brus-Ramer et al., 2007; Carmel et al., 2010, 2013, 2014). The model we chose is the unilateral pyramidal tract lesion, which we use for two reasons. First, unilateral pyramidal tract section allows unambiguous identification of the reaction of spared CST axons to injury. This lesion eliminates all CST axons from one hemisphere to the contralateral side of the spinal cord, thereby allowing us to examine explicitly spared axons from the unaffected hemisphere projecting to the affected and unaffected sides of the spinal cord; the ipsilateral and contralateral, respectively (**Figure 1B**). By making the lesion in the brain stem not the spinal cord, we can identify the reaction of spared CST axons to the loss of the majority of CST axons and to stimulation without the contributions of inflammatory and other reactions at the injury site. Second, the pyramidal tract lesion is also a rigorous model to assess the effects of stimulation on recovery mediated by the ipsilateral motor cortex and CST. This is relevant for large hemispheric stroke, and also for spinal cord injury, which usually spares only sparse descending motor connections.

We sought to determine the roles of injury- and activity-dependent plasticity in CST sprouting. We measured spinal axon terminations within the spinal cord on the same side as the anterograde injections of motor cortex. Terminations to this side of the spinal cord are sparse and come from either ipsilaterally descending connections, or double crossed connections (**Figure 1B**). Pyramidal tract lesion is a useful experimental approach, not only to model the response of the undamaged CST after a large unilateral injury, but also because the spared ipsilateral CST axons mimic the sparse innervation found after a paralyzing injury.

Our animal models use electrical stimulation of the CST in the medullary pyramid (Brus-Ramer et al., 2007) or, to facilitate translation to the human, motor cortex stimulation (Carmel et al., 2010, 2013, 2014). Either stimulation site leads to increased activity of a defined motor system, the corticofugal system, which includes direct spinal projections as well as cortical projections to the brain stem (Carmel et al., 2013). Corticospinal motor system stimulation contrasts with use of behavioral approaches, such as constraint-induced movement therapy (CIMT), which likely changes the activation patterns of multiple motor and somatic sensory pathways, as well as the primary afferent fibers (Mark et al., 2006). In our experiments we stimulate for 10 days. This time period was chosen on the basis of our developmental studies in which a 10-day to 2-week stimulation period was sufficient to produce significant augmentation of the CST spinal projection (Salimi and Martin, 2004; Salimi et al., 2008).

As in our development studies, we used stimulation parameters that activate the movement circuit from motor cortex to muscle. Motor cortex electrical stimulation may mimic the general increase in engagement and excitability that is experienced during high-intensity training. Thus we used a stimulus that produces phasic activation of contralateral forelimb muscle and often a small contralateral forelimb movement. The presence of a motor output ensures that spinal motor circuits, the targets of corticospinal axons, are activated. This contrasts with other studies that used phasic subthreshold stimulation (e.g., Wang et al., 2012), which may not activate the spinal cord.

Animals are stimulated in their home cage using implanted electrodes attached to a head connector. The train of stimuli that we used (16 biphasic pulses at 333 Hz of 0.2 ms duration) was found to optimally provoke movement during intracortical microstimulation. These stimulation parameters result in a motor response that, unlike some motor cortex stimulation protocols [e.g., theta burst stimulation (Huang et al., 2005; Cardenas-Morales et al., 2010)], does not increase motor evoked potentials. We applied this every 2 seconds, since the excitability of the CST returns to baseline by this time, for 6 hours a day. The stimulation is given during the day, when the rats normally sleep, and the rats will sleep during most of the stimulation period. We have observed no pain response or other adverse reactions to stimulation.

## HARNESSING NEURAL ACTIVITY-DEPENDENT PROCESSES AFTER INJURY IN MATURITY STRENGTHENS AND AUGMENTS SPROUTING OF SPARED CST AXONS

Stimulation of the corticospinal motor system for 10 days significantly augments the strength of the ipsilateral motor responses (Brus-Ramer et al., 2007). This was shown by conducting a terminal experiment after the 10-day stimulation period. We used a stimulating electrode over the surface of the intact pyramid and recorded contralateral and ipsilateral evoked responses in the deep radial nerve. This is the nerve that innervates many distal forelimb muscles, including those that are routinely activated by CST electrical stimulation. We computed the ipsilateral–contralateral response amplitude ratio, using the contralateral evoked response to control for depth of anesthesia and variability of efficacy in the stimulating electrodes.

**FIGURE 2:**
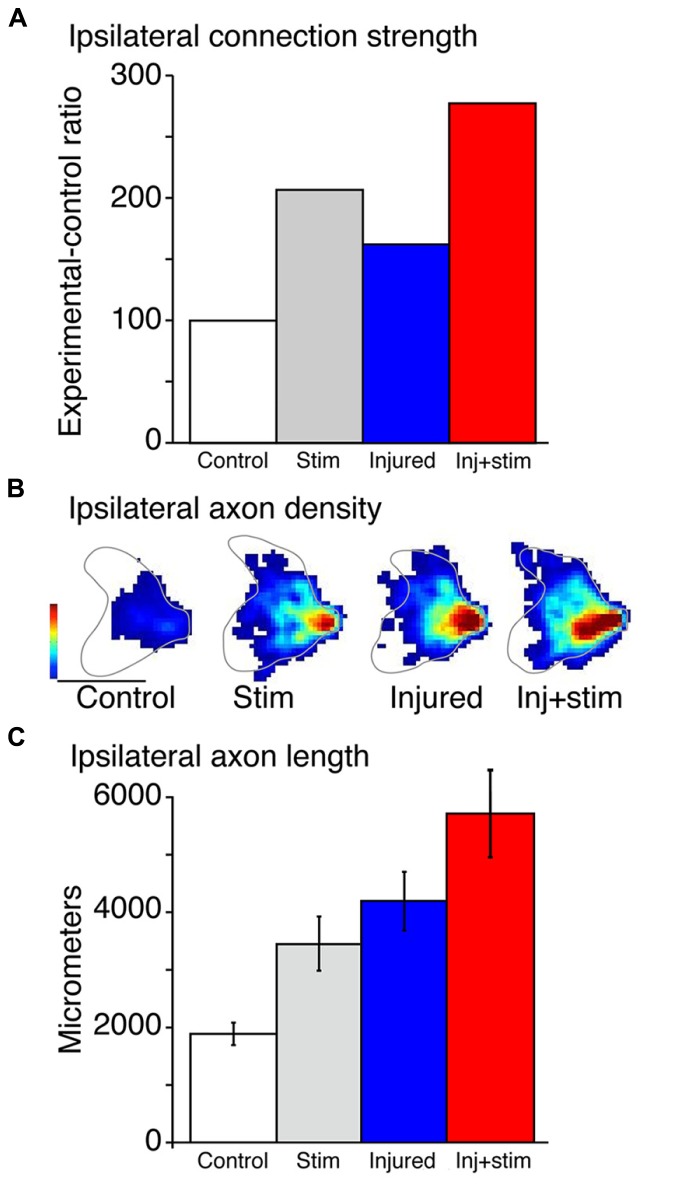
**Effects of stimulation, injury and their combination on the ipsilateral corticospinal tract. (A)** Changes in the strength of ipsilateral corticospinal tract axons are assayed by the ratio of the thresholds to evoke an ipsilateral response and a contralateral response in each animal. Stimulation (Stim) and injury alone both show enhanced capacity to evoke ipsilateral motor responses, which is augmented further in the combined condition (Inj + stim). **(B)** The regional density of corticospinal tract axons within the ipsilateral spinal cord is displayed as heatmaps for each condition. Each heat map is an average of 4–6 animals per group. The color scales are the same for all animals. Calibrations: 500 μm; Color scale: 0–5.7 μm axon/μm^2^ area. **(C)** Stimulation (Stim) and injury alone and in combination augment total ipsilateral CS termination axon length. Total average ipsilateral axon length in the controls, stimulation alone, injury alone, and combined injury and stimulation. Stimulation (*p* < 0.011) and injury (*p* < 0.003) alone each augmented total axon length significantly compared with control. Combined, there was a larger increase (*p* < 0.002). *p* Values were calculated from *t* test with Bonferroni/Dunn correction. **(A–C)** were modified from Brus-Ramer et al. (2007).

Using pyramidotomy, electrical stimulation, and their combination, we compared four groups of animals: naive rats, injury only, stimulation only, or stimulation after injury. To assess CST connection strength we electrically stimulated the pyramidal tract and recorded evoked responses in a forelimb nerve bilaterally. As a physiological assay, pyramidal tract stimulation is more selective for the CST than motor cortex stimulation; corticobulbar and corticorubral projections may be recruited with motor cortex stimulation. Furthermore, the difference between the contralateral and ipsilateral current thresholds is greater in the pyramid than motor cortex and can thus provide a more sensitive measure of ipsilateral connection strength. The current threshold for producing an evoked motor nerve potential in naïve rats is roughly 5 times the current amplitude than the threshold for eliciting a contralateral response. With stimulation alone, there is a strong augmentation of the ipsilateral response to stimulation (**Figure 2A**). This activity-dependent plasticity is similar in magnitude to the effects of injury-dependent plasticity in rats with injury only (**Figure 2A**; compare Stim and Injured bars). Stimulating the spared CST in injured animals (**Figure 2A**; Inj + Stim) augments the ipsilateral response more than either intact or injured-alone animals; the effects are additive. This shows the injury-dependent augmentation of CST spinal connections can be further enhanced by activity.

These changes in CST response efficacy are paralleled by changes in the local density of CST spinal terminations (**Figures 2B,C**). This is revealed using a different approach. An anterograde tracer is injected into motor cortex on the uninjured side before the stimulation period, and the distribution of labeled axons in the ipsilateral cervical enlargement is assessed the day after the 10-day stimulation period. The regional density of CST axons within the gray matter is plotted as a heat map (**Figure 2B**). The anatomical effects of injury- and activity-dependent plasticity mimic physiological changes; stimulation and injury each promote axon terminal sprouting and rats, with injury and stimulation showing an additive effect of each (compared **Figure 2C** with **Figure 2A**). Changes in the density of axon varicosities, which are putative synaptic boutons (Li and Martin, 2001) parallel those of axon termination density (Brus-Ramer et al., 2007).

Where are the sprouting axons and their boutons located within the spinal cord? We found that sprouting occurs largely within the normal territory of ipsilateral terminations, in the medial intermediate zone. This is a region containing last-order interneurons in the rodent spinal cord (Arber, 2012), including cholinergic interneurons that make C-boutons on the cell body of motoneurons. Thus, the increase in ipsilateral CST projection likely explains the observed increase in the strength of the evoked motor responses. We show below that stimulation also augments other corticofugal pathways, which may contribute to the strengthening of connections between the brain on the uninjured side and the ipsilateral spinal cord, which is the impaired side of the rat.

## ELECTRICAL STIMULATION OF THE SPARED MOTOR CORTEX HELPS RECONSTRUCT THE DAMAGED PATHWAY

Pyramidal tract lesion denervates the contralateral spinal cord through the loss of ~95% of the CST. Our developmental findings, which point to the importance of activity-dependent competition in early establishment of CST connections, suggest that the regions that have lost the most CST axons would gain the most with stimulation because there is less competition between sparse terminations compared with dense ones. To better understand how activity changes the distribution of CST axon terminations after injury, we measured the effects of motor cortex electrical stimulation within the ipsilateral and contralateral sides of the spinal cord; the affected (denervated) and unaffected sides, respectively. We hypothesized that stimulation would produce a more robust sprouting in the ipsilateral than the contralateral spinal cord.

**FIGURE 3:**
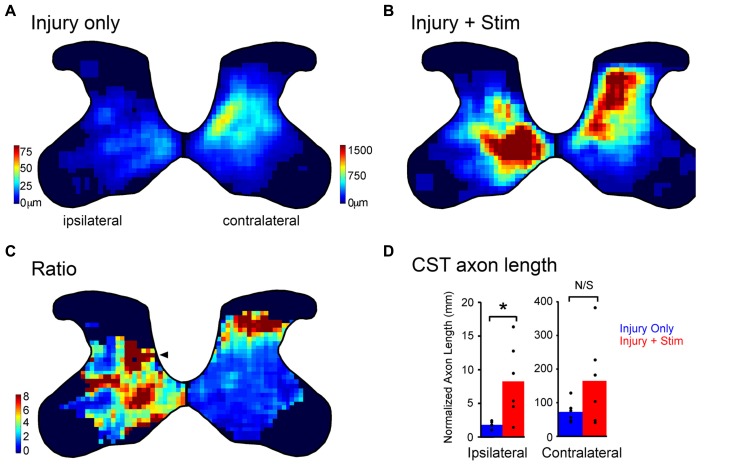
**Partial restoration of the normal axon distribution on the impaired side of the spinal cord. (A,B)** Heatmaps of corticospinal tract axon length for rats with injury only **(A)** and combined injury and stimulation **(B)**. Intensity scales in **(A)** also apply to **(B)**. Note, the axon length scales are different on the two sides to show the full range on each side. **(C)** Stimulation induced outgrowth is shown as the pixel-by-pixel ratio of injury and stimulation rats to injury only rats expressed as fold-change. The arrowhead indicates a hot spot of stimulation-induced outgrowth on the ipsilateral side in the deep dorsal horn. **(D)** Quantification of corticospinal axon length for the contralateral and ipsilateral sides (^*^p < 0.05). **(A–D)** were modified from Carmel et al. (2013).

The ipsilateral CST from one hemisphere does not terminate within the same territory as the contralateral CST from the other hemisphere. The ipsilateral CST is normally more focused within the medial two-thirds of the intermediate zone, whereas the contralateral CST projects throughout the deeper laminae of the dorsal horn and intermediate zone (**Figure 3A**, injury only). The total axon length in rats with injury only is ~40 times greater in the contralateral than ipsilateral spinal cord (**Figure 3D**; blue bars, note the different scales). We found that motor cortex electrical stimulation promoted outgrowth preferentially to the ipsilateral side of the spinal cord; the side that is largely denervated by the injury and is impaired. **Figure 3B** shows that injury plus stimulation yields a robust response both contralaterally and ipsilaterally. In terms of absolute change in CST axon length, there is a much greater amount on the contralateral side. However, the relative increase in ipsilateral relative to contralateral sprouting was striking: outgrowth was 4.6-fold in the spinal cord ipsilateral to stimulation and 2.3-fold greater to the contralateral side (**Figure 3D**), a statistically significant difference (Carmel et al., 2013). Thus, motor cortex stimulation drives outgrowth to both sides of the spinal cord, but outgrowth was stronger to the impaired side of the animal, which was denervated by the injury.

We hypothesized that axon outgrowth would be greatest in areas with low axon density because each stimulated axon could have less competition with other stimulated axons. This would explain the greater relative outgrowth to the sparsely innervated ipsilateral half of the spinal cord versus the densely innervated contralateral half. In addition, we hypothesized that competition would also help define the pattern of local axon outgrowth. To determine the pattern of stimulation-induced outgrowth we compared the axon distribution for rats with injury only (**Figure 3A**) to those with injury and stimulation (**Figure 3B**). **Figure 3C** shows the ratio of local pixel values in rats with injury and stimulation to those with injury only. On the ipsilateral side we see axon outgrowth that is particularly intense in the normal contralateral distribution pattern (arrowhead), suggesting reconstitution of the normally adaptive pattern of the lost contralateral CST innervation. On the contralateral side greatest outgrowth was located dorsal to the normally innervated region in the middle of the dorsal horn. Thus, we found that stimulated CST axons behave according to similar competition rules we observed during development. Importantly, this results in a preferential strengthening of circuits on the impaired side of the spinal cord in a pattern that is adaptive for limb control.

**FIGURE 4:**
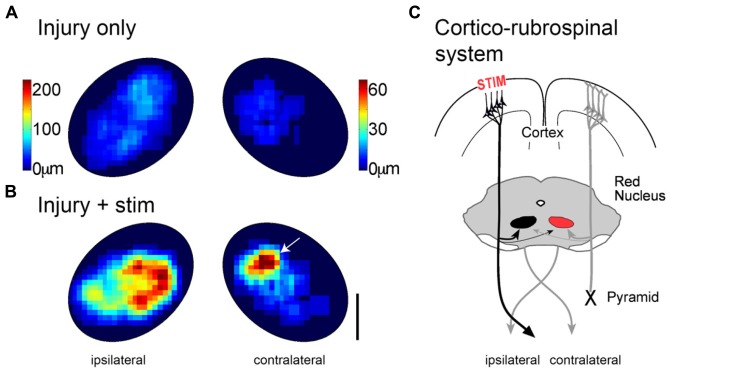
**Motor cortex stimulation causes robust outgrowth to the magnocellular red nucleus.** Heatmaps showing corticorubral axon density in the magnocellular red nucleus after injury alone **(A)** and injury plus stimulation **(B)**. Color scales apply to both **(A,B)**. Note the axon length scales are different on the two sides to show the full range on each side. **(C)** Schematic organization of corticorubral system and stimulation. Outgrowth to the contralateral nucleus (right) could improve motor control on the impaired side through the re-crossed rubrospinal tract. Modified from Carmel et al. (2013).

We also tested the effects of cortical stimulation on outgrowth to important brain stem targets. We were particularly interested in determining if motor cortex stimulation augmented corticorubral outgrowth into the magnocellular red nucleus, which gives rise to the rubrospinal tract (see **Figure 4C**). Like the spinal cord, motor cortex stimulation produced massive (3- to 5-fold) increase in axon density (**Figures 4A** and **4B**). In addition, we observed a selective increase in the density of corticorubral axon terminations in the presumed forelimb region of the contralateral magnocellular red nucleus (**Figure 4B**; arrow), which projects to the affected side of the spinal cord. Thus, coordinated outgrowth in the spinal cord and red nucleus could support partial re-establishment of motor systems connections to the denervated spinal motor circuits. Selective inactivation of each of these pathways will be important to determine the relative contribution of each to the reparative effects of motor cortex stimulation.

## MOTOR CORTEX STIMULATION OF THE INTACT CST AFTER PYRAMIDOTOMY RESTORES SKILLED FORELIMB MOVEMENT

**FIGURE 5:**
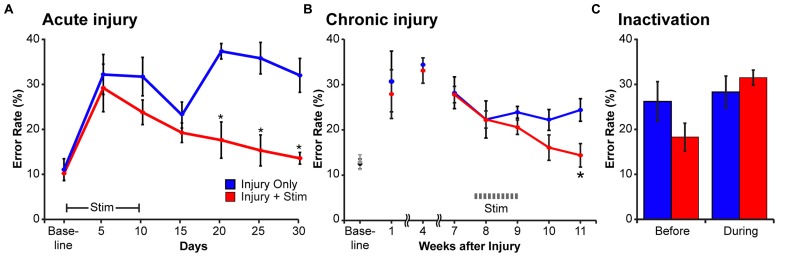
**The stimulated motor cortex mediates motor recovery after injury.** Rats were trained to cross a horizontal ladder with irregularly spaced rungs until they achieved a baseline error rate below 20%. **(A)** Effect of motor cortex stimulation beginning the day after injury. The error rates increased in the affected forelimb to a similar degree in rats with injury only (blue) and rats with injury and stimulation (red). Modified from Carmel et al. (2010). **(B)** Effect of motor cortex stimulation after chronic injury. Until the start of stimulation (weeks 1–7) the error rates in the two groups were not different. After the start of stimulation (weeks 8–11) the groups differed significantly (repeated measures ANOVA, with Bonferroni *post hoc* correction, asterisk, *p* = 0.03). **(C)** After stimulation, motor cortex inactivation reinstates the motor impairment. After completion of motor cortex stimulation, performance on the horizontal ladder was measured before and during inactivation. In the rats with injury only, inactivation did not change the error rate in the impaired forelimb (blue bars are not different). In contrast, in rats with injury and stimulation (red bars) inactivation of the stimulated motor cortex reinstated their initial deficit in the ipsilateral forelimb (paired *t*-test, *p* = 0.01). **(B,C)** were modified from Carmel et al. (2014).

Having demonstrated that CST electrical stimulation can strengthen connections with spinal motor circuits and promote axon outgrowth, we next determined if the injured animals showed improved motor recovery after stimulation. We tested CST function using a horizontal ladder with irregularly spaced rungs. To perform the task correctly, the rat needs to integrate sensory information about the position of the next rung (visual, vibrissal, and somatosensory) and alter the trajectory of the step to place the paw accurately on the rung. This sensory-motor transformation is a key attribute of the corticospinal system (Porter and Lemon, 1993). After training to a criterion error rate, rats had a pyramidotomy and the next day began 10 days of electrical stimulation using the protocol described above. We measured task performance every 5 days for 30 days (**Figure 5A**). Rats with injury only had a persistent deficit in the forelimb contralateral to pyramidotomy. By contrast, rats with injury and motor cortex stimulation had a significant reduction in forelimb errors (Carmel et al., 2010). At end of testing, the performance of stimulated animals was not different from uninjured rats. Interestingly, in each of the six animals examined there was transient improvement on day 15 that was followed by a worsening of performance on the next examination day. The improvement may be due to early injury-dependent sprouting (Brus-Ramer et al., 2007) that is not maintained without motor cortex stimulation (Carmel et al., 2010). The types of errors that the rats made (understep of the rung, overstep, or miss) were not different from baseline, suggesting recovery of neurological function rather than behavioral compensation (Krakauer et al., 2012). Importantly, we never observed maladaptive effects of stimulation. There was consistent improvement on the affected side, ipsilateral to stimulation and performance on the unaffected side remained stable (Carmel et al., 2010).

We also asked whether stimulation could improve motor function after chronic injury. This question has clear implications for translation, since most patients are chronically injured. It also raises an important neurobiological question: does activity, in the form of electrical stimulation, have to be added at the time when injury-induced plasticity is highest, as suggested by some training studies (Biernaskie et al., 2004), or is it equally effective in chronic injury? We tested the efficacy of motor cortex electrical stimulation 8 weeks after pyramidotomy. Like rats with stimulation after acute injury, those with chronic injury had a full restoration of skilled locomotion with electrical stimulation (Carmel et al., 2014). Stimulation was delivered 8 weeks after injury and by 11 weeks, performance was no different from baseline (**Figure 5B**). Similar to our earlier anatomical study (Brus-Ramer et al., 2007), this suggests independent and complementary roles for activity- and injury-dependent plasticity.

## A DELAYED RECOVERY PROGRAM

There is a curious parallel between the anatomical and behavioral effects of CST electrical stimulation: they each show greatest changes in the period after stimulation has ended. In the case of spinal axon sprouting, when we examined axon sprouting the day after the stimulation period, rats with injury and stimulation had 35% more axon length in the spinal cord ipsilateral to stimulation compared with injury only rats (**Figure 2**; Brus-Ramer et al., 2007). By contrast, when we examined the axon length at the end of the behavior testing period, which was 30 days after stimulation ended, the rats with stimulation had 460% more axon length than rats with injury only (Carmel et al., 2013). Similarly, behavioral changes become manifest after the stimulation period after both acute (**Figure 5A**) and chronic (**Figure 5B**) injury.

What could account for the delayed behavioral response, which we hypothesize is mediated by CST outgrowth? We stimulate the CST near its cell body. If axons sprout using mRNA or proteins from the cell body, these would need to be transported to the terminations, which takes time. For the behavior, it could take time for rats to incorporate the stronger ipsilateral circuits into their motor repertoire. What could account for the substantially greater outgrowth observed 30 days after stimulation than at 10 days? We hypothesize that stimulation sets off a growth program that lasts beyond the stimulation period. In support of this hypothesis is that sprouting persists 30 days after cessation of stimulation and, at this late time point, the amount of sprouting is substantially greater than at 10 days. This could put the animal on a heightened trajectory for recovery, whereby the stronger ipsilateral connections are used more effectively in skilled limb control. This enhanced performance, in turn, could lead to greater activation and further outgrowth.

## PATHWAYS FOR MOTOR RECOVERY

We used reversible inactivation (Martin and Ghez, 1999) to determine if the stimulated motor cortex was responsible for recovery after chronic CST injury. At least five weeks after stimulation, we microinjected the GABA_A_ agonist muscimol into the simulated motor cortex and determined changes in motor performance of the ipsilateral forelimb. For healthy controls, motor cortex inactivation does not produce an ipsilateral motor impairment, only contralateral impairments (Carmel et al., 2014). For injury only animals, motor cortex inactivation does not significantly exacerbate errant control (**Figure 5C**, blue bars). This suggests that injury-dependent sprouting is insufficient to mediate recovery via the ipsilateral CST. By contrast, in injured plus stimulated animals motor cortex inactivation reinstated the impairment to a level that was not different from performance immediately after the pyramidal tract lesion (**Figure 5C**, red bars). Importantly, once the effects of the muscimol inactivation wore off, behavioral recovery returned (data not shown).

Taking our anatomical and behavioral findings together, motor recovery begins in the stimulated motor cortex. The most likely pathway is the augmented direct ipsilateral CST. As there is substantial outgrowth to the contralateral spinal cord, we propose that recovery could be aided by the contralateral CST through connections to spinal commissural circuits. Finally, the highly targeted corticorubral projections to the contralateral magnocellular red nucleus (**Figure 4B**) could provide stronger drive to the rubrospinal tract serving the affected limb. We suggest that the remarkable and complete skilled locomotor recovery after injury plus stimulation is made possible by coordinated sprouting of the stimulated motor cortex to several of its subcortical targets. Although skilled locomotion is highly dependent on the CST, and is thus a good assay for promoting corticospinal motor system function, it remains to be determined if motor cortex stimulation substantially improves reaching and manipulation.

## CONSIDERATIONS FOR TRANSLATION TO THE HUMAN WITH SCI

Our experiments with rats show that there is a durable recovery mediated by motor cortex stimulation. We showed that there is abundant sprouting of spared CST axons. Stimulation to promote CST sprouting may be distinguished from the use of repetitive TMS-based approaches, such as intermittent theta burst stimulation (Huang et al., 2005), which is thought to produce control improvements through physiological changes (Thickbroom, 2007). The possibly of achieving a more durable improvement through structural plasticity would justify the use of more invasive approaches, such as motor cortex epidural stimulation. Importantly, motor cortex stimulation promoted motor recovery after both acute and chronic injury, dispelling the notion that there is a critical period for activity-based intervention after CST injury. This has important implications for devising treatments in chronic spinal cord injury and stroke.

A critical advance will be to show efficacy of motor cortex stimulation in a animal model of SCI without production of maladaptive effects. Anatomical and electrophysiological outcomes need to be evaluated to determine if stimulation is sufficiently robust to augment sprouting above or below the injury site. In the context of SCI, the optimal stimulation parameters need to be determined and, if effective, the range of motor behaviors that can be improved by motor cortex stimulation. As stimulation promotes sprouting, it will be important to determine in a spinal injury model if there are any maladaptive effects. Does CST sprouting reduce hyperreflexia by increasing descending modulation of spinal reflexes (Ahmed, 2014), or might it worsen hyperreflexia observed after injury (Tan et al., 2012) by increasing spinal excitability? It is important to note that in our studies, sprouting into the side of the spinal cord with normal function did not alter performance of unimpaired limbs (Carmel et al., 2010). Indeed, it is heartening that sprouting of descending motor connections has not resulted in spasticity or pain, either in our experiments or others.

For translation, should we adopt an invasive stimulation approach, as in our rodent epidural cortical stimulation studies, or a non-invasive approach? We chose invasive stimulation because of its ability to selectively activate forelimb area of motor cortex, as evidenced by production of forelimb but not hind limb or whisker responses. In humans, epidural and subdural electrodes have been used safely for monitoring before epilepsy surgery, pain control, and to promote motor recovery after stroke. Besides stimulating selective areas of cortex, invasive stimulation allows long periods of stimulation either at rest or during motor training without specialized equipment. This approach has already been used successfully: when the presence of spared CST axons is taken into account, motor stimulation can promote significant behavioral improvement (Nouri and Cramer, 2011). Thus, in people with spinal cord injury or stroke we must identify the presence of a spared corticospinal projection as an essential condition for using this approach. The obvious disadvantages of this approach are the risk to the patient, and the cost and complexity of the neurosurgery involved.

In parallel, we need to determine if non-invasive stimulation approaches are capable of achieving CST sprouting, strengthening of connections, and improved motor recovery. Of the non-invasive approaches, transcranial magnetic stimulation (TMS) has similar biophysics to the invasive stimulation that we have used; each produce phasic depolarization of the CST. In the rat, we have been limited in trying this approach because TMS activated brain stem motor pathways directly (Nielsen et al., 2007), and not just motor cortex. However, TMS methods are being developed to more selectively activate rat motor cortex (Gasca et al., 2010; Rotenberg et al., 2010). Selective targeting with TMS is less likely to be a problem for humans. Transcranial direct current stimulation may also be useful in promoting CST function, although this may be through its effects on motor learning (Reis et al., 2009; Fritsch et al., 2010). In translating to the human, it is important to recognize that the principle rule of this approach is to promote sprouting and strengthen connections of spared CST axons.

Our work can be interpreted in the context of recent exciting results that spinal epidural stimulation can promote voluntary movement in people with chronic spinal cord injury (Harkema et al., 2011; Angeli et al., 2014). Spinal epidural stimulation increases the excitatory drive of spinal motor circuits and can allow weak descending control to become manifest (Roy et al., 2012; Borton et al., 2013). Our work demonstrates that motor cortex stimulation can strengthen the weak descending circuits that provide the substrate for recovery of volitional movement. Thus, the two approaches act in complementary ways to promote motor control – by strengthening weak descending motor control in the case of motor cortex stimulation, and by increasing receptiveness to these control signals in the case of spinal cord stimulation. Although we postulate that these are the main mechanisms through which these stimulation techniques work, it is interesting to note that CST innervation itself can act to modify spinal excitability (Brus-Ramer et al., 2009) and spinal stimulation can increase descending motor control, when it is paired with rehabilitation (Musienko et al., 2012). Pairing of motor cortex and spinal cord stimulation could be a powerful approach to restoring corticospinal function (Nishimura et al., 2013). Indeed, pairing of brain and peripheral stimulation, which is thought to recruit spinal circuits, can help restore function in rodents (Ahmed, 2013) and people (Bunday and Perez, 2012) with spinal cord injury.

There are very promising strategies for reconnecting the brain and spinal cord after SCI, but we are far from achieving that goal. Particularly intriguing are recent genetic approaches to reprogram damaged neurons to facilitate axonal outgrowth (Liu et al., 2010); and in particular, to regenerate lost axons. Such manipulations to augment axonal outgrowth, combined with viral delivery methods that achieve cell-specificity, could be very effective. Success and safe implementation in humans will no doubt take many years of development. While very promising, brain machine interfaces (BMI) and assistive approaches do not, and may not for the foreseeable future, offer the necessary motor outcomes or independence that patients need.

As we discussed, activity-based interventions tap into well-established mechanisms for formation and specification of neural connections. Unlike systemic treatments, electrical stimulation can be targeted to specific circuits within the nervous system. This has the potential to be more efficacious for the circuits targeted for repair and also to have fewer adverse effects on non-targeted brain regions or other organ systems. Empirically, activity-based approaches are safe and are well-tolerated procedures in animals and humans. Most important, they offer relative ease of translation to the human compared with genetic and biochemical approaches.

To conclude, axon regeneration has proven to be a more difficult problem to solve than we thought 20 years ago. Promoting sprouting of spared spinal motor systems, and training new connections to mediate adaptive functions, is one of the few – arguably the only – circuit-based approach on the horizon. By optimizing electrical stimulation to promote CST function in animals, we can sharpen our understanding of its mechanisms. This new understanding can then be translated quickly to test similar hypotheses in people with spinal cord injury. Thus, we can leverage our understanding of the specific neural circuits that mediate behavior to selectively reinforce critical connections and restore function after injury.

## Conflict of Interest Statement

The authors declare that the research was conducted in the absence of any commercial or financial relationships that could be construed as a potential conflict of interest.
